# Promoting socioemotional development in early childhood: implementation and evaluation of the VIPP-SD parenting intervention in Portugal

**DOI:** 10.1186/s40359-025-03431-3

**Published:** 2025-10-29

**Authors:** Manuela Veríssimo, Maryse Guedes, Marilia Fernandes, Carla Fernandes, Carolina Santos, Eva Diniz, Paula Oliveira, Mariana Negrão, Filipa Sampaio, Marian Bakermans-Kranenburg

**Affiliations:** 1 William James Center for Research (WJCR), ISPA-Instituto Universitário, Lisboa, Portugal; 2 ProChild CoLAB, Guimarães, Portugal; 3 Universidade Católica Portuguesa, Faculty of Education and Psychology, Research Centre for Human Development, Port, Portugal; 4 Department of Public Health and Caring Sciences, Uppsala University, Uppsala, Sweden; 5 Facultad de Psicología y Humanidades, Universidad San Sebastián, Sede Valdivia, Chile

**Keywords:** VIPP-SD, Parenting Intervention, Socioemotional Development

## Abstract

**Background:**

The prevention of internalizing and externalizing behavioral problems in children is a critical scientific and public health priority. Research highlights maternal sensitivity—defined as a caregiver’s ability to perceive, interpret, and respond appropriately to their child’s cues—and consistent but non-coercive discipline as key factors in reducing these behavioral issues. The Video-feedback Intervention to Promote Positive Parenting and Sensitive Discipline (VIPP-SD) aims to enhance maternal sensitivity and promote non-coercive discipline strategies. Meta-analyses have demonstrated its effectiveness in improving parental sensitivity, limit-setting practices, and child attachment security, particularly among socioeconomically disadvantaged families. However, evidence on its impact on externalizing behaviors remains mixed, with some studies suggesting delayed or context-specific effects. This project aims to evaluate VIPP-SD’s impact on parental sensitivity, discipline, child behavioral problems, and parental mental health in Portugal. Additionally, it seeks to assess the intervention’s cost-effectiveness by analyzing health outcomes, resource utilization, and associated costs.

**Methods:**

The program consists of a baseline visit, four intervention sessions, and optional booster sessions, focusing on themes such as sensitive responsiveness, positive reinforcement, and empathetic boundary-setting. 120 families from vulnerable populations in Portugal will be recruited and randomly assigned to the VIPP-SD intervention or the same number of contacts without feedback on parenting. Trained interveners will deliver the intervention. Pre- and posttest assessments include observed and self-reported parenting behaviors, parental mental health, quality of life, and resource use. Follow-up assessments include questionnaires on parent and child variables.

**Discussion:**

The study aims to provide robust evidence to inform health policy decisions and prioritize cost-effective early interventions that improve developmental outcomes, reduce societal costs, and support family well-being.

**Trial registration:**

NCT07153198, 02/09/2025.

**Supplementary Information:**

The online version contains supplementary material available at 10.1186/s40359-025-03431-3.

Given the high prevalence of internalizing and externalizing behavioral problems among youth, their prevention represents an important scientific and public health priority [3, 34]. Empirical evidence indicates that increasing maternal sensitivity—defined as a caregiver’s ability to perceive, interpret, and respond appropriately to their child’s cues—may be an effective strategy for reducing the incidence and severity of these behavioral problems over time [8, 13, 27]. Therefore, interventions aimed at enhancing maternal sensitivity could play a crucial role in promoting better developmental outcomes and mitigating future behavioral difficulties in children.

In addition to its potential to improve developmental outcomes, the prevention of internalizing and externalizing behavioral problems may also yield significant economic benefits. By reducing the incidence and severity of these issues through interventions that increase maternal sensitivity, it is possible to decrease the long-term costs associated with mental health services, educational support, and other related resources. Thus, investing in preventive strategies not only supports the well-being of children and families but may also contribute to substantial savings in healthcare and social service expenditures in the future [32]. In line with Heckman’s research, which shows that investments in early childhood education provide higher economic returns compared to later-stage interventions, such investments increase productivity, reduce social costs, and help alleviate social inequality by giving disadvantaged children a better start [16].

## VIPP-SD

The Video-feedback Intervention to Promote Positive Parenting and Sensitive Discipline (VIPP-SD) is an attachment- and social learning-based intervention aimed at promoting parental sensitivity and the use of developmentally appropriate, non-coercive discipline strategies [18]. The VIPP-SD intervention integrates the enhancement of parental sensitive responsiveness [2] with parent coaching focusing at preventing coercive parent-child interaction cycles [24] and facilitating sensitive limit-setting practices. VIPP-SD consists of a baseline visit and four intervention sessions (with the option of one or two additional booster sessions), held weekly or biweekly, depending on the family's needs and availability. A previous meta-analysis revealed that intensive interventions with numerous sessions often revealed negative effect sizes, in contrast with shorter and more focused interventions [5].

The VIPP-SD approach aims at strengthening parenting skills and promoting a sensitive and responsive caregiving environment, fostering secure parents-child attachment and better socio-emotional outcomes, with a lower likelihood of long-term behavioral problems. Specifically, VIPP-SD addresses disciplinary strategies alongside sensitivity themes through: (1) the differentiation between attachment and exploration behaviors; interpretation of child's behavior and significance of a sensitive response to it; and the importance of sharing emotions; (2) the application of distraction and induction as non-coercive techniques in response to challenging child behaviors or conflict-prone situations; (3) the utilization of positive reinforcement, whereby parents praise adaptive behaviors and ignore maladaptive, attention-seeking actions; (4) the implementation of a sensitive interaction pause to de-escalate conflict or temper tantrums; and (5) the demonstration of empathy towards the child in conjunction with consistent disciplinary approaches and explicit boundary-setting [18]. Two booster sessions are meant to reinforce and consolidate all intervention themes.

Developed and tested for over 30 years with more than 2,000 families in 25 RCTs, VIPP-SD [18, 38] has been found to substantially reduce the prevalence of insecure and disorganized attachment in early childhood and is associated with lower levels of externalizing behavior problems in children in several recent studies [23, 31]. A recent meta-analysis [38] concluded that the VIPP-SD intervention had significant positive effects on parenting behavior and attitudes, promoting parental sensitivity and limit setting across various groups, regardless of psychological status. Through increased parental sensitivity and discipline, the intervention was found to improve child attachment security. No overall significant effect was found on reducing child externalizing behavior, although some studies suggest effects may appear over time or in specific contexts. The largest, most robust pragmatic trial to date found a positive effect on externalizing behaviors for at-risk children [23].

In Portugal, a prior randomized controlled trial was performed with socioeconomically disadvantaged families who struggled with multiple stress factors. VIPP-SD was effective in enhancing positive parent-child interactions and positive family relations [22]. The findings also revealed that the VIPP-SD program was successful in reducing maternal harsh discipline among mothers who initially reported high levels of parenting stress. These results underscore the program’s potential to enhance parenting practices in families that are more vulnerable to harsh discipline and child maltreatment [25]. Contemporary science recognizes the importance of replication studies. This project builds on previous work by incorporating measures of sensitivity, parental anxiety and depression, and in addition aims to demonstrate the economic impact of intervening in early relational experiences.

The main objective of the present project is to evaluate whether the VIPP-SD program contributes to enhancing parental sensitivity and promoting the adoption of sensitive discipline parenting practices, with the ultimate goal of reducing behavioral problems in children and improving parental mental health outcomes. Furthermore, the study aims to assess the costs and cost-effectiveness of the VIPP-SD intervention. By analyzing its impact on health outcomes, resource utilization, and associated costs, this research seeks to highlight the potential economic advantages of early intervention. The findings may provide critical evidence to support health policy decision-makers in prioritizing cost-effective preventive strategies for early intervention.

### Objectives and hypotheses

1. The primary objective of the current project is to examine the effect of the VIPP-SD on parental sensitivity and sensitive discipline of the target parent, from baseline to 8 weeks after and 6 months after the intervention. We expect a statistically significant increase in sensitivity and sensitive discipline among parents from the intervention condition (VIPP-SD) when compared with parents from control condition ("dummy" contacts with interveners) from pre- to post-intervention.
2. The secondary aim of the study is to examine the value of the intervention in reducing children’s behavioral problems, as a function of increased parental sensitivity and the use of sensitive discipline strategies. It is expected that improvements in parental sensitivity and sensitive discipline resulting from the VIPP-SD intervention will be associated with lower levels of behavioral difficulties in intervention group children when compared with children from control condition ("dummy" contacts with interveners) from pre- to post-intervention and follow-up intervention.
3. A third objective is to evaluate the effects of the VIPP-SD on parent-reported parenting practices, parental anxiety and depression symptoms, and parental health related quality of life. We expect that parents in the intervention condition will report a decrease in depression and anxiety symptoms, as well as more nurturing and less restrictive parenting practices from pre- to post-intervention and 6-months follow-up.
4. The fourth objective is to examine the cost-effectiveness of the intervention by assessing the differential impact of the intervention compared to the control on quality adjusted life years (QALY) and resource use and related costs. We expect that VIPP-SD leads to improved health related quality of life (HRQoL) and a reduction in the use of societal resources compared to the control condition.

## Methods

### Recruitment

One hundred and twenty families will be recruited from families attending Family Support and Parental Counseling Centers (CAFAP) and Child Psychology Centers in the Metropolitan area of Lisbon. CAFAP is a specialized social response aimed at supporting families with children and adolescents who are in situations of vulnerability, risk, or danger. CAFAP operates through referrals from entities such as Child and Youth Protection Commissions (CPCJ), schools, health services, or social security, with all interventions conducted in agreement with the legal guardians.

A minimum of 60 families in each condition will participate in the study, with an equal balance (through stratified randomization) of boys and girls. Inclusion criteria for parents will be: (1) fluency in Portuguese, (2) having a child between the ages of 2 and 6 years, and (3) absence of any diagnosed psychopathology, chronic or hereditary illnesses. Parents of children with a known diagnosis of disabilities, chronic or hereditary illnesses will be excluded from the study, since they are eligible for other intervention programs that are targeted to their specific needs. The expected completion date for participant recruitment is July 31, 2026.

We conducted a Power Analysis to ensure sufficient statistical power, based on previous VIPP studies. Expected effect size for primary outcome is meta-analytically *f* = 0.18 [38]. Assuming *f* = .18, power = .90, and *α* = .05, and correlation between pretest and posttest of *r* = .35, at least 54 participants per group are required. To account for attrition, we set a target of 60 per group.

### Intervention

In the present study, the VIPP-SD protocol will be implemented using a culturally adapted and linguistically validated Portuguese version [21]. All materials were translated into Portuguese, using the committee approach outlined by [6]. This method involved multiple bilingual translators to ensure the accuracy and cultural relevance of the content. Additionally, all intervention practitioners were trained and will be supervised by certified VIPP-SD trainers, ensuring that they met the standards required for effective implementation of the intervention.

All intervention practitioners participating in the study received formal training in the VIPP-SD protocol. The training was conducted by certified VIPP-SD professionals and adhered to the official guidelines to ensure consistency and fidelity to the intervention model.

To ensure fidelity, intervention feedback scripts will be coded for content based on the guidelines outlined in a coding fidelity manual. To support this process, random sessions will be videotaped or audiotaped for review.

### Study design

**Fig. 1:**
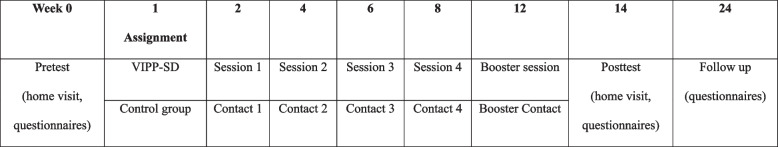
Schedule of the design, sessions, and assessments

To evaluate the intervention’s effectiveness, we will conduct a two-arm randomized controlled trial (RCT) in which participating families will be randomly assigned to either an intervention group or a control group ("dummy" contacts with interveners) after the baseline visit. Parents in the Control Group will receive, at the same frequency as the Intervention Group’s sessions, a phone call addressing topics related to child development (e.g., sleep, nutrition, etc., but not providing feedback on parenting). The protocols will be specifically designed to encourage participants to talk about their children. Randomization will be performed using a computer-generated random allocation sequence, stratified by child age and sex to ensure balanced group characteristics and group assignment will be done after the baseline assessment is completed to minimize potential bias (Fig. 1).

### Instruments

Data collection for sociodemographic variables will take place at baseline (pretest). Parental sensitivity and sensitive discipline will be coded from videotaped mother–child interactions by independent, trained observers who are blinded to intervention conditions; these measures will be collected at baseline and immediately post-intervention (posttest). All other measures will be collected at three time points: baseline, posttest, and follow-up. Due to time and personnel limitations, the follow-up evaluation will rely solely on questionnaires.

#### Parental sensitivity and sensitive discipline (observation)

Parental sensitivity will be assessed using the the revised Erickson 7-point rating scales for Supportive Presence (from 1 = *parent completely fails to be supportive to the child* to 7 = *parent skilfully provides support throughout the session*) and Intrusiveness (from 1 = *parent allows the child sufficient time to explore and to attempt to solve tools on her/his own* to 7 = *parent is highly intrusive; her agenda clearly has precedence over the child’s wishes*; [7]. These scales are age-adequate adaptations of the Ainsworth Maternal Behavior Scales [2], which are widely regarded as a gold standard for evaluating the sensitive responsiveness of parents to their children's cues. Supportive presence is defined as the consistent emotional, physical, and psychological availability of a mother, characterized by both verbal expressions and nonverbal behaviors. High sensitivity is associated with positive developmental outcomes in children, including secure attachment and better emotional regulation. Intrusiveness reflects the extent to which a parent disregards or interferes with the child’s autonomy, wishes, interests, or behaviors. The scale captures behaviors that may disrupt the child’s ability to express themselves or make independent decisions. Intrusiveness is often characterized by over-controlling or overly directive parenting, and it can negatively impact the child’s development, particularly their sense of autonomy and emotional well-being. Both sensitivity and intrusiveness are crucial dimensions of the parent-child interaction and provide valuable insights into the quality of the parenting style.

Parental discipline will be observed during a compliance task that tends to elicit challenging child behavior. Three scales will be coded: Positive Limit-Setting, using the revised Erickson 7-point rating scales for Supportive Presence (from 1 = *parent completely fails to provide positive limit-setting*, to 7 = *parent skillfully provides positive limit-setting*; [7], Physical Interference, rated on a 5-point scale (from 1 = *parent does not interfere physically*, to 5 = *parent often interferes physically*; [39], and Laxness, reflecting inconsistent or lenient approaches to setting and enforcing limits, rated on a 5-point rating scale (from 1 = *no laxness* to 5 = *continuous laxness*; [36].

#### Strengths & Difficulties Questionnaire (SDQ, [12] Portuguese version: Fleitlich et al. 10

This 25-item parent-rated scale evaluates emotional and behavioral problems in children aged 2 to 17 years. The items are rated on a 3-point scale: 0 = "*Not true*," 1 = "*A bit true*," and 2 = "*Very true*." The questionnaire is organized into five subscales: (1) emotional symptoms, (2) conduct problems, (3) hyperactivity, (4) peer problems, and (5) prosocial behavior. The total difficulties score is derived by summing the first four subscales (excluding prosocial behavior), yielding a score ranging from 0 to 40. Studies with Portuguese children have reported internal consistency values ranging from 0.60 to 0.70 for the Parent version [1].

#### Child Rearing Practices Report–Questionnaire (CRPR-Q)

The *Child Rearing Practices Report–Questionnaire* (CRPR-Q; [30] is a 40-item parent-report questionnaire used to assess parenting practices that are theoretically and empirically linked to child developmental outcomes. Parents are instructed to respond with reference to a specific child in the household, rating each item on a 6-point Likert scale ranging from 1 (*strongly disagree*) to 6 (*strongly agree*). The measure comprises two theoretically grounded subscales: Nurturance, which captures parenting behaviors characterized by warmth, emotional availability, and expressions of affection (e.g., “I express affection by hugging, kissing, and holding my child”); Restrictiveness, which reflects parental emphasis on behavioral control, obedience, and caution in risk-taking (e.g., “I prefer that my child not try things if there is a chance he will fail”). Mean scores are calculated for each subscale, with higher values indicating greater levels of the respective parenting behavior. These dimensions are considered critical for understanding variability in children’s socioemotional, behavioral, and cognitive development. In the Portuguese adaptation of the CRPR-Q, the instrument demonstrated satisfactory internal consistency, with Cronbach’s alpha coefficients of .83 for Nurturance and .86 for Restrictiveness [28], supporting its reliability for use in developmental research within Portuguese-speaking populations.

#### Depression, Anxiety, and Stress Scale – 21 (DASS-21)

The *Depression, Anxiety, and Stress Scale – 21* (DASS-21; [19] is a 21-item self-report questionnaire designed to assess symptoms of depression, anxiety, and stress in parents. Each item is rated on a 4-point Likert scale ranging from 0 (*Did not apply to me at all*) to 3 (*Applied to me most of the time*). The scale comprises three subscales: Depression (7 items), which assesses dysphoria, hopelessness, devaluation of life, self-deprecation, lack of interest or involvement, anhedonia, and inertia; Anxiety (7 items), which measures autonomic arousal, skeletal muscle effects, situational anxiety, and the subjective experience of anxious affect; Stress (7 items), which evaluates chronic, nonspecific arousal, including difficulty relaxing, nervous arousal, irritability, agitation, and impatience. Subscale and total scores are calculated by summing the item responses. Both the original and Portuguese versions of the DASS-21 have demonstrated strong internal consistency, Cronbach’s alpha coefficients were 0.90 for the depression subscale, 0.86 for anxiety, 0.88 for stress, and 0.95 for the total score combining the three subscales, and construct validity [4].

#### Resource use

Resources used by children and their parents will be measured using a questionnaire adapted from published questionnaires [26]. Resource use will include healthcare services (accident and emergency visits, primary care and specialized outpatient visits and inpatient stays, medication), social services, transportation, absenteeism and presenteeism from both paid and unpaid work, and out-of-pocket expenses. This measure will be used for economic evaluation.

#### AQOL-8D: Assessing Quality of Life - 8 Dimensions [29]

Quality of life among caregivers will be measured with the Assessing Quality of Life - 8 Dimensions (AQOL-8D; [29] is a multi-attribute utility instrument developed to provide a sensitive and comprehensive assessment of health status among adults across eight dimensions,, including independent living, mental health, coping, relationships, pain, self-worth, happiness and senses) and 35 items with four to six response levels, each representing increasing levels of severity. This measure will be used for economic evaluation.

#### Evaluation of the intervention

Participants allocated to the VIPP-SD intervention will be invited to complete a feedback questionnaire upon the completion of the program. The evaluation of participant satisfaction will focus on their overall experience with the intervention, including their perceptions of the effectiveness of VIPP-SD and the suitability of the treatment setting.

#### Sociodemographic questionnaire

A Sociodemographic Questionnaire will be administered to collect background information on both the child and their parents. This will include data such as sex, age, nationality, household composition, socioeconomic status, and educational level.

### Data analysis

Data inspection procedures will be performed once the data collection is completed, but prior to the commencement of formal statistical analyses. These preliminary analyses will focus on examining the quality and integrity of the data collected, identifying any potential errors, outliers, or inconsistencies. Data validation steps will include checking for missing values, verifying the distribution of key variables, and ensuring adherence to predefined inclusion criteria. The results of this inspection will guide decisions on data cleaning, imputation, or any necessary adjustments before proceeding with more advanced statistical methods.

For all study objectives, the impact of the VIPP-SD intervention will be compared to the control condition using intent-to-treat (ITT) analyses. To assess the intervention's effect in the key outcomes, including parental sensitivity and sensitive discipline, child behavior problems (SDQ), depression, anxiety, and stress (DASS-21) and child rearing practices (CRPR-Q), we propose the use of a repeated measure ANOVA. In this model, experimental condition will be treated as a between-subjects factor, and assessment time-point as a within-subjects factor. The regression coefficient for the interaction between condition and assessment time will estimate differential changes in parental sensitivity and sensitive discipline between the intervention and control groups over time.

### Health economic evaluation

The health economic evaluation will follow the Consolidated Health Economic Evaluation Reporting Standards 2022 Statement [17] and will include a series of cost-utility analyses using cost per quality adjusted life years (QALYs) gained for children (using SDQ scores mapped onto a multi-attribute utility instrument using published algorithms), for caregivers (using the scores from the AQOL-8D) and for the dyad as the outcome. Costs will be analyzed from two perspectives: a healthcare system perspective (the Portuguese National Health Service; SNS) and a broader societal perspective. The healthcare system perspective includes the cost to run the intervention, collected from project documentation. The broader societal perspective includes, in addition to intervention costs, other healthcare costs (healthcare utilization and medication), as well as costs beyond healthcare, including social services, transportation, out-of-pocket expenses, and productivity losses related to absenteeism and presenteeism from paid and unpaid work. Total costs and QALYs will be aggregated over the trial period and estimated using the area under the curve approach [20]. Differences in total QALYs and costs for children and caregivers will be analyzed using regression models. Incremental cost-effectiveness ratios (ICER) will be estimated as the ratio between the difference in costs and the difference in QALYs for children, caregivers and the dyad for each costing perspective. Uncertainty around the cost and QALY estimates will be explored using non-parametric bootstrapping and plotted on cost effectiveness planes [11]. The probability of cost-effectiveness of the intervention at different values of willingness to pay will be represented visually as cost-effectiveness acceptability curves [9].

### Ethics

The project was submitted and approved by the ISPA Ethics Committee (I-011-04-25). All procedures performed in the current study will be in accordance with the recommendations of APA Ethical Guidelines, and with the 1964 Helsinki declaration and its later amendments, ensuring the privacy and confidentiality of participants’ information.

The project will involve volunteer human adults (parents) and vulnerable minor participants. Service technicians will be approached and asked to disseminate study materials and invitations to potential participants. Aims and procedures will be explained to parents. Participants will be informed that they can decline or withdraw at any time without any consequences, and that their decision not to take part in the study will in no way affect the care, support, or follow-up services they and their families received from the referring agencies. Parents will be asked for written informed consent and children's dissent will be respected. Participation in this project will be entirely voluntary. The risks for participants will be minimized by collecting only necessary data and by scheduling interventions with minor interference in participants' routines. The project will collect the strictly necessary sensitive personal data. Participants will be assigned unique identifiers (only known by the researcher). Identifying data will be stored on a computer that is not connected to the internet, to ensure participant confidentiality. Statistical analyses will only be conducted with files containing alphanumeric identifiers. Informed consent and collected data containing identifying information will be stored under lock, separately from data identified by a study code number. The project will involve video-recording sessions that will be conducted at home. Confidentiality and privacy will be ensured by storing informed consent and data containing identifying information (video recordings) under lock and key, separately from data identified by a study code number. Electronic databases and archives will be protected by passwords. Video recordings will be delivered to the families and destroyed after 10 years of the project.

If suspicion or evidence of maltreatment is observed during home visits, families will be referred to the appropriate authorities or organizations for further evaluation and intervention.

### Data sharing

Personal data will not be shared due to concerns about confidentiality and legal restrictions. Sharing data could risk exposing proprietary or sensitive information, including details that may affect the entire family’s privacy. Data sharing on the aggregate level will be possible via the principal investigator.

### Advisory board

The study is supported by an advisory board consisting of both parents and health care professionals, who provide expert-by-experience guidance to the research and intervention team, following the Cooperative Practitioners-Researchers Model [37]. The advisory board recommends the best practices for communicating with the target population, particularly with respect to materials such as flyers and phone calls. Their input ensures that communication strategies are appropriately tailored to the needs and preferences of the target group. Additionally, the advisory board offers recommendations on cultural sensitivity, accessibility of information, outreach strategies, and engagement with community organizations.

## Discussion

Investments in early childhood are very important and yield greater economic and social returns compared to interventions made later in life [15]. This is particularly critical in Portugal, where the prevalence of mental health disorders among youth, especially anxiety and depression, is rising. According to the WHO-backed HBSC survey conducted in 2022, 27.7 % of Portuguese adolescents reported feeling unhappy, a substantial increase from 18.3 % in 2018 (Health Behaviour in School-aged Children (HBSC), 14. In this context, several randomized controlled trials have demonstrated the efficacy of the original VIPP intervention and its adaptations, reinforcing the robust theoretical framework of the VIPP-SD program [38]. The present study is thus timely and significant, aiming to enhance understanding of early preventative interventions that foster emotional and behavioral regulation, mitigate the onset of mental health problems, and reduce long-term societal and economic burdens. This parenting intervention is expected to improve parental sensitivity and more effective discipline techniques, which, in turn, should promote better developmental outcomes for children. This could result in fewer behavioral problems and a decrease in depression and anxiety symptoms among the caregivers involved [35].

The large number of interveners may introduce some variability in the implementation of the intervention. However, through supervision and team meetings and weekly discussion, we aim to minimize this variability across participants and ensure that the program is delivered consistently.

The use of observation and real-time data collection techniques offers a precise and relatively objective assessment of parenting behaviors, minimizing the biases often found in self-report questionnaires [33]. This approach not only strengthens the reliability of the findings but also provides a deeper, more nuanced understanding of how the intervention impacts participants over time, offering valuable insights into the dynamics of real-life interactions and behavioral change. A key advantage of the study will be estimating the economic value of the intervention, utilizing Quality-Adjusted Life Years (QALYs) as a metric for evaluating outcomes. By addressing both developmental and economic outcomes, this study provides decision-makers with a robust framework for assessing the cost-effectiveness of the VIPP-SD intervention, and for evaluating whether improvements in parents' and children’s well-being justify the resources invested.

Providing parents with the tools and support to prevent harsh discipline and promote positive interactions during early years helps create a healthier, more supportive environment for children. This proactive approach significantly reduces the risk of maltreatment and emotional challenges later in life and may be more cost-effective than waiting until these issues have fully developed and become harder to treat.

## Supplementary Information

Supplementary Material 1.

## Data Availability

Not applicable.
